# Risk model for morbidity and mortality following liver surgery based on a national Japanese database

**DOI:** 10.1002/ags3.12803

**Published:** 2024-04-16

**Authors:** Tatsuya Orimo, Shinya Hirakawa, Akinobu Taketomi, Hisateru Tachimori, Taro Oshikiri, Hiroaki Miyata, Yoshihiro Kakeji, Ken Shirabe

**Affiliations:** ^1^ Department of Gastroenterological Surgery I Hokkaido University Graduate School of Medicine Sapporo Japan; ^2^ Database Committee The Japanese Society of Gastroenterological Surgery Tokyo Japan; ^3^ Endowed Course for Health System Innovation Keio University School of Medicine Tokyo Japan; ^4^ Department of Healthcare Quality Assessment, Graduate School of Medicine The University of Tokyo Tokyo Japan; ^5^ Department of Health Policy and Management Keio University School of Medicine Tokyo Japan; ^6^ The Japanese Society of Gastroenterological Surgery Tokyo Japan

**Keywords:** hepatectomy, liver resection, morbidity, mortality, risk model

## Abstract

**Aim:**

We evaluated the morbidity and mortality associated with liver surgery in Japan and developed a risk model for liver resection using information from a national database.

**Methods:**

We retrospectively reviewed 73 861 Japanese patients who underwent hepatectomy between 2014 and 2019, using information from the National Clinical Database (NCD) registrations. The primary endpoints were 30 days and in‐hospital mortality, and the secondary endpoints were postoperative complications. Logistic regression risk models for postoperative morbidity and mortality after hepatectomy were constructed based on preoperative clinical parameters and types of liver resection, and validated using a bootstrapping method.

**Results:**

The 30‐day and in‐hospital mortality rates were 0.9% and 1.7%, respectively. Trisectionectomy, hepatectomy for gallbladder cancer, hepatectomy for perihilar cholangiocarcinoma, and poor activities of daily living were statistically significant risk factors with high odds ratios for both postoperative morbidity and mortality. Internal validations indicated that the c‐indices for 30‐day and in‐hospital mortality were 0.824 and 0.839, respectively.

**Conclusions:**

We developed a risk model for liver resection by using a national surgical database that can predict morbidity and mortality based on preoperative factors.

## Graphical abstract

We evaluated the morbidity and mortality associated with liver surgery and developed a risk model for liver resection by using information from the National Clinical Database.

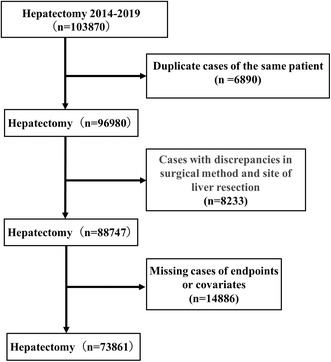

## 1 INTRODUCTION

Liver resection is an important curative treatment option for various liver tumors, including hepatocellular carcinoma, intrahepatic cholangiocarcinoma, metastatic liver tumors, gallbladder cancer, and perihilar cholangiocarcinoma, among others. These surgeries were originally very high‐risk but have become safer in recent years. The Nationwide Inpatient Sample (NIS) database from the United States indicated that the overall mortality rate from hepatic resection declined from 10.4% in 1989 to 5.3% in 2000.^1^ The mortality rate has been reported to be approximately 2%–3% in recent years.^2^

Large databases have become popular for analyzing the perioperative risks of different surgeries. The American College of Surgeons manages the large NSQIP (National Surgical Quality Improvement Program) database, which produces results of this nature across various surgical fields, including risk analysis of liver resection.^2^, ^3^ The National Clinical Database (NCD) in Japan was developed in collaboration with the NSQIP, with the common goal of creating a standardized surgical database for quality improvement. NCD data have now yielded morbidity and mortality results for various cancer surgeries.^4^, ^5^, ^6^, ^7^, ^8^, ^9^ Analyses of risk models for liver resection that can be applied nationally have also been performed to date using NCD data.^10^, ^11^ Notably, however, these analyses have been based on 1 or 2 years of data and are limited to certain types of liver resection in the study population.

Therefore, there is a need for longer‐term and more comprehensive analyses.

In the present study, we examined more than 70 000 liver resections over a 6‐year period at centers across Japan and performed a risk analysis of these operations based on information from the NCD. We further developed risk models for hepatectomy to improve the quality of these procedures. This study is the largest in the world to date on risk modeling of liver resection.

## 2 METHODS

### 2.1 Data collection

**FIGURE 1:**
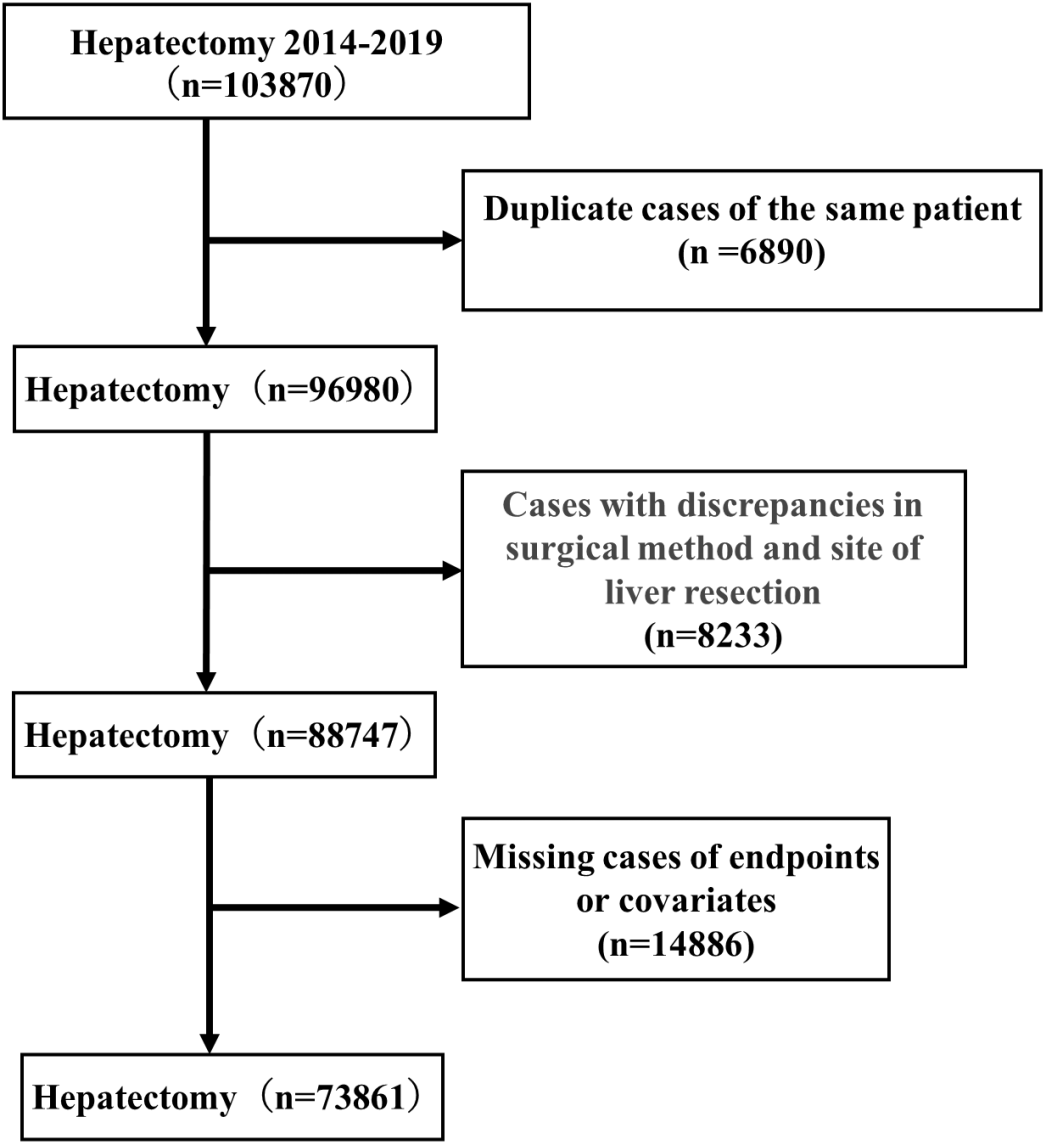
Study population flow chart.

This data used in this study were obtained from the NCD, a nationwide collaborative project established in April 2010, involving the Japanese surgical board certification system. More than 5500 facilities participate in the NCD, and information on approximately 1.5 million cases is collected annually. The NCD is currently the world's largest clinical database linked to a medical specialty system. We retrospectively collected data from the NCD for liver resections conducted between 2014 and 2019. These cases included partial liver resection, segmentectomies 1–8, left lateral sectionectomy, right anterior sectionectomy, right posterior sectionectomy, right hepatectomy, left hepatectomy, central bisectionectomy, right trisectionectomy, left trisectionectomy, hepatectomy for gallbladder cancer, and hepatectomy for perihilar cholangiocarcinoma. All hepatectomies for gallbladder cancer or perihilar cholangiocarcinoma were classified as either hepatectomy for gallbladder cancer or hepatectomy for perihilar cholangiocarcinoma, even if a right or left hepatectomy, or a right or left trisectionectomy, was performed. After excluding duplicate procedures for the same patient, cases with discrepancies in the surgical method and site of liver resection, and cases with missing endpoints and covariates, 73 861 patients were finally included in the present study population (Figure 1). The Ethics Committee of the NCD approved the retrospective use of data collected by the NCD for observational research.

### 2.2 Endpoints

The primary endpoints of this study were 30‐day mortality and in‐hospital mortality, defined as death within 30 days of surgery, regardless of whether the patient was in hospital or out of hospital, and death within the period of hospitalization, including 30‐day mortality, respectively. Secondary endpoints included postoperative complications, such as reoperation, surgical site infection (organ space), bile leakage, pneumonia, renal failure, postoperative blood transfusion, and sepsis.

### 2.3 Statistical analysis

Descriptive statistics were generated for the demographic, clinical, and laboratory characteristics of the study subjects. For continuous variables, the median and the 25th and 75th percentile values were calculated. Categorical variables were expressed as numbers and percentages. Risk models were developed for each outcome using backward stepwise logistic regression with Akaike's Information Criterion (AIC). Candidate covariates for inclusion in the risk models were selected from registry variables related to outcomes according to previous studies and clinical findings.^10^, ^11^ Regression coefficients and intercepts, odds ratios (ORs), 95% confidence intervals (CIs), and P values for each coefficient were calculated for the final logistic regression model. The performance of the risk model was evaluated using a c‐index and a calibration plot. Optimism of the c‐index was corrected using Harrell's bias correction method. CIs for the c‐index were calculated using the location‐shifted bootstrap method because the sample size was sufficiently large.^12^ The number of bootstrap samples was set at 200. Bootstrapping was used to correct for optimism in the calibration plot. The statistical significance level was set at 0.05, and all tests were two‐tailed.

All statistical analyses were performed using R software version 4.1 and later (R Foundation, Vienna, Austria, https://www.r‐project.org/). The backward stepwise variable selection method and calibration plots were performed using the rms R package version 6.2 and later (Frank E Harrell Jr., https://CRAN.R‐project.org/package=rms).

## 3 RESULTS

### 3.1 Risk profile of the study population

The clinical features of the study cohort are presented in Table 1. The liver resections in our current series included partial and anatomical resections, such as segmentectomy, one sectionectomy, two sectionectomy, trisectionectomy, hepatectomy for gallbladder cancer, and hepatectomy for perihilar cholangiocarcinoma. The top three primary diagnoses included in this study were hepatocellular carcinoma in 30 093 (40.7%) patients, metastatic liver tumor in 22 726 (30.8%) patients, and intrahepatic cholangiocarcinoma in 5748 (7.8%) patients. The abbreviated risk profiles and preoperative laboratory data for the study population are shown in Table 1. Briefly, 12.4% of the patients had a history of preoperative chemotherapy within 90 days prior to surgery, 1.8% had a history of weight loss >10%, and 3.8% had a body mass index >30 kg/m^2^. The identified pre‐existing comorbidities were as follows (frequency in parentheses): diabetes mellitus (internal medicine therapy) (17.1%), diabetes mellitus (insulin therapy) (5.9%), heavy alcohol use (30.5%), chronic obstructive pulmonary disease (3.9%), hypertension (40.6%), preoperative dialysis (0.9%), previous cerebrovascular disease (3.4%), chronic steroid use (1.0%), and anticoagulant therapy (6.0%). The American Society of Anesthesiologists (ASA) classification of III to V was assigned in 13.2% of the cases.

**TABLE 1:** Clinical characteristics of the entire study cohort.

Variables	Value (*n* = 73 861)	%
Age	70 (63–76)	
Gender		
Female	23 831	32.3
Male	50 030	67.7
Type of liver resection		
Partial resection	26 795	36.3
Segmentectomy 1	389	0.5
Segmentectomy 2	257	0.3
Segmentectomy 3	647	0.9
Segmentectomy 4	2285	3.1
Segmentectomy 5	1232	1.7
Segmentectomy 6	1056	1.4
Segmentectomy 7	886	1.2
Segmentectomy 8	2306	3.1
Left lateral sectionectomy	4181	5.7
Right anterior sectionectomy	3182	4.3
Right posterior sectionectomy	4854	6.6
Right hepatectomy	8588	11.6
Left hepatectomy	8323	11.3
Central bisectionectomy	1261	1.7
Right trisectionectomy	483	0.7
Left trisectionectomy	388	0.5
Hepatectomy for gallbladder cancer	1489	2.0
Hepatectomy for perihilar cholangiocarcinoma	5259	7.1
Primary diagnosis		
Hepatocellular carcinoma	30 093	40.7
Intrahepatic cholangiocarcinoma	5748	7.8
Metastatic liver tumor	22 726	30.8
Gallbladder cancer	2062	2.8
Perihilar cholangiocarcinoma	4947	6.7
Others	8790	11.9
Preoperative risk assessment		
Ambulance transport	570	0.8
Emergency operation	421	0.6
Preoperative chemotherapy	9131	12.4
Preoperative radiotherapy	432	0.6
Body mass index >30 kg/m^2^	2828	3.8
Diabetes (internal medicine therapy)	12 598	17.1
Diabetes (insulin therapy)	4328	5.9
Current smoker (within 1 year)	14 366	19.5
Alcoholism	22 550	30.5
Preoperative respiratory distress	69	0.1
ADL full support within 30 d before surgery	195	0.3
Ventilator dependent	56	0.1
COPD	2868	3.9
Pneumonia	110	0.1
Hypertension	29 978	40.6
Congestive heart failure	245	0.3
Previous myocardial infarction within 6 m	209	0.3
Angina within 30 d before surgery	665	0.9
Previous PCI	2034	2.8
Previous cardiac surgery	874	1.2
Acute renal failure	61	0.1
Dialysis	661	0.9
Previous cerebrovascular disease	2528	3.4
Disseminated cancer	2626	3.6
Chronic steroid use	708	1.0
Weight loss >10%	1322	1.8
Anticoagulant therapy	4407	6.0
Preoperative blood transfusion	517	0.7
Sepsis	170	0.2
ASA classification >3	9782	13.2
Preoperative laboratory data		
Platelets <150 000/μL	16 221	22.0
Albumin <4.0 g/dL	30 458	41.2
Total bilirubin >1.2 mg/dL	6119	8.3
AST >35 U/L	19 810	26.8
ALT >35 U/L	16 977	23.0
ALP >340 U/L	20 620	27.9
PT% <70 or PT sec >12	8402	11.4

Abbreviations: AL, Activities of daily living; ASA, American Society of Anesthesiologists; COPD, chronic obstructive pulmonary disease.

### 3.2 Morbidities and mortalities after liver resection

The morbidity and mortality rates in the study cohort are shown in Table 2. The 30‐day mortality rate was 0.9%, and the in‐hospital mortality rate was 1.7%. With respect to postoperative complications, the rate of reoperation was 2.8%, surgical site infection was 5.0%, bile leakage was 7.3%, pneumonia was 1.7%, renal failure was 1.7%, postoperative blood transfusion was 4.3%, and sepsis was 2.0%.

**TABLE 2:** Morbidities and mortalities among the NCD hepatectomy study population (*n* = 73 861).

Outcomes	Overall incidence, *n* (%)
30‐day mortality	665 (0.9%)
In‐hospital mortality	1291 (1.7%)
Reoperation	2076 (2.8%)7
Surgical site infection (Organ space)	3657 (5.0%)
Bile leakage	5372 (7.3%)
Pneumonia	1223 (1.7%)7
Renal failure	1247 (1.7%)
Postoperative blood transfusion	3196 (4.3%)7
Sepsis	1514 (2.0%)

### 3.3 Risk models for 30‐day mortality and in‐hospital mortality

Logistic regression risk models for postoperative mortality after hepatectomy were constructed based on the preoperative clinical parameters and types of liver resection. Table 3 presents the results. The mortality risk was calculated as follows:

$$\text{Predicted mortality}=1/\left(1+\exp\left(-\left(\text{β0}+\beta 1\text{X1}+\beta 2\text{X2}+\ldots +\text{βnXn}\right)\right)\right)$$

where β0 is the intercept and β1, β2, …, βn are the coefficients corresponding to the predictor variables X1, X2, …, Xn, respectively.

**TABLE 3:** Risk models for 30 days mortality and in‐hospital mortality.

Outcome	Risk factor	Coefficient	*p* value	Odds ratio	95% CI
30‐day mortality	Intercept	−10.02	<0.001	0	0.00–0.00
	Age	0.40	<0.001	1.50	1.36–1.64
	Gender (female)	Reference	–	–	–
	Gender (male)	0.38	<0.001	1.46	1.21–1.77
	No ambulance transport	Reference	–	–	–
	Ambulance transport	0.58	0.02	1.79	1.10–2.93
	No emergency operation				
	Emergency operation	0.74	0.015	2.09	1.15–3.78
	Body mass index ≤30 kg/m^2^	Reference	–	–	–
	Body mass index >30 kg/m^2^	0.33	0.105	1.39	0.93–2.08
	No diabetes	Reference	–	–	–
	Diabetes (no treatment)	0.49	0.062	1.63	0.98–2.74
	Diabetes (dietary treatment only)	0.19	0.424	1.21	0.76–1.91
	Diabetes (internal medicine therapy)	0.16	0.124	1.17	0.96–1.42
	Diabetes (insulin therapy)	0.42	0.004	1.52	1.15–2.01
	ADL Independence	Reference	–	–	–
	ADL partial support just before surgery	0.29	0.121	1.33	0.93–1.91
	ADL full support just before surgery	1.04	<0.001	2.83	1.53–5.24
	No angina	Reference	–	–	–
	Angina within 30 d before surgery	0.55	0.046	1.72	1.01–2.94
	No history of cardiac surgery	Reference	–	–	–
	Previous cardiac surgery	0.46	0.064	1.58	0.97–2.57
	No disseminated cancer	Reference	–	–	–
	Disseminated cancer	0.54	0.006	1.71	1.17–2.51
	No weight loss	Reference	–	–	–
	Weight loss >10%	0.61	<0.001	1.83	1.29–2.60
	No anticoagulant therapy	Reference	–	–	–
	Anticoagulant therapy	−0.24	0.116	0.79	0.58–1.06
	No preoperative blood transfusion	Reference	–	–	–
	Preoperative blood transfusion	0.41	0.141	1.50	0.87–2.58
	PLT ≥150 000/μL	Reference	–	–	–
	PLT <150 000/μL	0.48	<0.001	1.61	1.34–1.94
	ALB ≥4.0 g/dL	Reference	–	–	–
	ALB <4.0 g/dL	0.25	0.008	1.28	1.07–1.54
	AST ≤35 U/L	Reference	–	–	–
	AST >35 U/L	0.42	<0.001	1.52	1.29–1.79
	ALP ≤340 U/L	Reference	–	–	–
	ALP >340 U/L	0.55	<0.001	1.73	1.44–2.07
	Creatinine male:0.61–1.04 mg/dL, female:0.47–0.79 mg/dL	Reference	–	–	–
Creatinine male	>1.04 mg/dL or <0.61 mg/dL; female >0.79 mg/dL or <0.47 mg/dL	0.35	<0.001	1.42	1.20–1.67
	Serum Na 138–146 mEq/L	Reference	–	–	–
	Serum Na >146 or < 138 mEq/L	0.16	0.127	1.17	0.96–1.44
	CRP ≤0.1 mg/dL	Reference	–	–	–
	CRP >0.1 mg/dL	0.32	<0.001	1.38	1.15–1.66

	PT INR ≤1.1	Reference	–	–	–
	PT INR >1.1	0.31	0.007	1.36	1.09–1.70
	PT% ≥70 or PT sec ≤12	Reference	–	–	–
	PT% <70 or PT sec >12	0.35	0.002	1.42	1.14–1.78
	No history of cerebrovascular disease	Reference	–	–	–
	Previous cerebrovascular disease	0.26	0.13	1.30	0.93–1.82
	ASA classification 1 and 2	Reference	–	–	–
	ASA classification ≥3	0.46	<0.001	1.59	1.31–1.92
	Partial resection	Reference	–	–	–
	Segmentectomy	−0.11	0.657	0.90	0.56–1.43
	Left lateral sectionectomy	0.20	0.448	1.22	0.73–2.04
	One sectionectomy	0.70	<0.001	2.01	1.48–2.72
	Two sectionectomy	1.06	<0.001	2.89	2.25–3.72
	Trisectionectomy	1.82	<0.001	6.16	3.87–9.80
	Hepatectomy for gallbladder cancer	1.59	<0.001	4.90	3.08–7.80
Hepatectomy for perihilar cholangiocarcinoma	2.09	<0.001	8.09	6.15–10.63
In‐hospital mortality	Intercept	−9.6	<0.001	0	0.00–0.00
	Age	0.38	<0.001	1.46	1.36–1.56
	Gender (female)	Reference	–	–	–
	Gender (male)	0.30	<0.001	1.35	1.17–1.55
	Body mass index ≤30 kg/m^2^	Reference	–	–	–
	Body mass index >30 kg/m2	0.28	0.063	1.33	0.98–1.79
	No diabetes	Reference	–	–	–
	Diabetes (no treatment)	0.30	0.155	1.35	0.89–2.06
	Diabetes (dietary treatment only)	0.09	0.627	1.09	0.77–1.55
	Diabetes (internal medicine therapy)	0.18	0.014	1.20	1.04–1.38
	Diabetes (insulin therapy)	0.35	<0.001	1.43	1.16–1.75
	ADL independence	Reference	–	–	–
	ADL partial support just before surgery	0.53	<0.001	1.70	1.33–2.17
	ADL full support just before surgery	1.04	<0.001	2.82	1.73–4.58
	No disseminated cancer	Reference	–	–	–
	Disseminated cancer	0.57	<0.001	1.77	1.34–2.33
	No weight loss	Reference	–	–	–
	Weight loss >10%	0.53	<0.001	1.70	1.31–2.21
	No preoperative blood transfusion	Reference	–	–	–
	Preoperative blood transfusion	0.53	0.008	1.70	1.15–2.51
	No sepsis	Reference	–	–	–
	Sepsis	0.86	<0.001	2.36	1.44–3.89
	HCT male ≥37; female ≥32	Reference	–	–	–
	HCT male <37; female <32	0.26	<0.001	1.29	1.14–1.47
	PLT ≥150 000/μL	Reference	–	–	–
	PLT <150 000/μL	0.46	<0.001	1.58	1.38–1.81
	ALB ≥4.0 g/dL	Reference	–	–	–
	ALB <4.0 g/dL	0.37	<0.001	1.45	1.26–1.67
	AST ≤35 U/L	Reference	–	–	–

	AST >35 U/L	0.43	<0.001	1.v53	1.36–1.73
	ALP ≤340 U/L	Reference	–	–	–
	ALP >340 U/L	0.50	<0.001	1.64	1.44–1.87
	Creatinine male:0.61–1.04 mg/dL, female:0.47–0.79 mg/dL	Reference	–	–	–
	Creatinine male >1.04 mg/dL or <0.61 mg/dL; female >0.79 mg/dL or <0.47 mg/dL	0.26	<0.001	1.30	1.15–1.47
	Serum Na 138–146 mEq/L	Reference	–	–	–
	Serum Na >146 or < 138 mEq/L	0.38	<0.001	1.46	1.26–1.68
	CRP ≤0.1 mg/dL	Reference	–	–	–
	CRP >0.1 mg/dL	0.24	<0.001	1.27	1.12–1.46
	PT INR ≤1.1	Reference	–	–	–
	PT INR >1.1	0.40	<0.001	1.49	1.27–1.75
	PT% ≥70 or PT sec ≤12	Reference	–	–	–
	PT% <70 or PT sec >12	0.26	0.002	1.29	1.10–1.52
	ASA classification 1 and 2	Reference	–	–	–
	ASA classification ≥3	0.49	<0.001	1.64	1.43–1.88
	No tumor or benign tumor	Reference	–	–	–
	Malignant tumor	0.32	0.052	1.38	1.00–1.91
	No respiratory distress	Reference	–	–	–
	Preoperative respiratory distress	0.73	<0.001	2.08	1.49–2.90
	Partial resection	Reference	–	–	–
	Segmentectomy	0.31	0.041	1.37	1.01–1.85
	Left lateral sectionectomy	0.27	0.163	1.31	0.90–1.92
	One sectionectomy	0.84	<0.001	2.33	1.86–2.91
	Right hepatectomy	1.39	<0.001	4.01	3.26–4.93
	Left hepatectomy	0.88	<0.001	2.42	1.90–3.08
	Central bisectionectomy	0.90	<0.001	2.45	1.58–3.81
	Right trisectionectomy	1.95	<0.001	7.04	4.66–10.63
	Left trisectionectomy	2.37	<0.001	10.67	6.92–16.46
	Hepatectomy for gallbladder cancer	1.78	<0.001	5.93	4.25–8.28
Hepatectomy for perihilar cholangiocarcinoma	2.14	<0.001	8.52	6.94–10.46

*Note*: Age is the value divided by 10.

Abbreviations: ADL, Activities of daily living; ASA, American Society of Anesthesiologists; CI, confidence interval; COPD, chronic obstructive pulmonary disease.

Statistically significant risk factors with an odds ratio of three times or more for 30‐day mortality included trisectionectomy (OR = 6.16), hepatectomy for gallbladder cancer (OR = 4.90), and hepatectomy for perihilar cholangiocarcinoma (OR = 8.09). Those for in‐hospital mortality were right hepatectomy (OR = 4.01), right trisectionectomy (OR = 7.04), left trisectionectomy (OR = 10.67), hepatectomy for gallbladder cancer (OR = 5.93), and hepatectomy for perihilar cholangiocarcinoma (OR = 8.52). The major risk factors for hepatectomy‐related mortality identified in this study were massive major hepatectomy and liver resection with biliary reconstruction. Among patient‐side factors, a risk factor for both 30‐day and in‐hospital mortality with a high odds ratio was full assistance with activities of daily living (ADL) within 30 days before surgery (OR = 2.83 for 30‐day mortality and OR = 2.82 for in‐hospital mortality).

### 3.4 Risk models for morbidities

Logistic regression risk models for postoperative morbidities were also constructed based on preoperative clinical parameters and types of liver resection and are presented in Table 4. Morbidity was calculated in the same way as mortality. With regard to postoperative complications, hepatectomy procedure was a statistically significant risk factor with a high odds ratio, as was mortality. Our present analyses further showed that, as well as risk factors for hepatectomy‐related mortality, trisectionectomy, hepatectomy for gallbladder cancer, or hepatectomy for perihilar cholangiocarcinoma could be risk factors for a variety of postoperative complications with odds ratios of three or more times. Patient‐side risk factors with odds ratios of three or more were ADL full support within 30 days of surgery, preoperative pneumonia for pneumonia, preoperative sepsis for renal failure, and preoperative sepsis for sepsis.

**TABLE 4:** Risk models for postoperative morbidities.

Outcome	Risk factor	Coefficient	*p* value	Odds ratio	95% CI
Reoperation	Intercept	−4.63	<0.001	0.01	0.01–0.01
	Gender (female)	Reference	–	–	–
	Gender (male)	0.23	<0.001	1.25	1.13–1.39
	No ambulance transport	Reference	–		
	Ambulance transport	0.38	0.034	1.46	1.03–2.08
	ADL Independence	Reference	–	–	–
	ADL partial support just before surgery	0.21	0.1	1.24	0.96–1.59
	ADL full support just before surgery	0.54	0.039	1.72	1.03–2.87
	No angina	Reference	–	–	–
	Angina within 30 d before surgery	0.41	0.028	1.51	1.04–2.17
	No disseminated cancer	Reference	–	–	–
	Disseminated cancer	0.54	<0.001	1.71	1.40–2.10
	No weight loss	Reference	–	–	–
	Weight loss >10%	0.38	0.002	1.46	1.15–1.86
	No anticoagulant therapy	Reference	–	–	–
	Anticoagulant therapy	−0.17	0.075	0.84	0.70–1.02
	No sepsis	Reference	–	–	–
	Sepsis	0.48	0.069	1.62	0.96–2.72
	Hemoglobin male ≥13.5 g/dL; female ≥11.5 g/dL	Reference	–	–	–
	Hemoglobin male <13.5 g/dL; female <11.5 g/dL	0.12	0.059	1.13	1.00–1.27
	HCT ≥ 37(male) or 32(female)	Reference	–	–	–
	HCT male <37; female <32	0.17	0.007	1.19	1.05–1.35
	PLT ≥150 000/μL	Reference	–	–	–
	PLT <150 000/μL	0.10	0.086	1.10	0.99–1.24
	ALB ≥4.0 g/dL	Reference	–	–	–
	ALB <4.0 g/dL	0.15	0.004	1.17	1.05–1.30
	ALT ≤35 U/L	Reference	–	–	–
	ALT >35 U/L	0.14	0.007	1.15	1.04–1.27
	ALP ≤340 U/L	Reference	–	–	–
	ALP >340 U/L	0.23	<0.001	1.26	1.14–1.40
	Creatinine male:0.61–1.04 mg/dL, female:0.47–0.79 mg/dL	Reference	–	–	–
Creatinine male	>1.04 mg/dL or <0.61 mg/dL; female >0.79 mg/dL or <0.47 mg/dL	0.08	0.111	1.09	0.98–1.20
	Serum Na 138–146 mEq/L	Reference	–	–	–
	Serum Na >146 or < 138 mEq/L	0.10	0.137	1.11	0.97–1.27
	CRP ≤0.1 mg/dL	Reference	–	–	–
	CRP >0.1 mg/dL	0.09	0.073	1.09	0.99–1.21
	PT INR ≤1.1	Reference	–	–	–
	PT INR >1.1	0.23	0.001	1.26	1.10–1.46
	PT% ≥70 or PT sec ≤12	Reference	–	–	–
	PT% <70 or PT sec >12	0.16	0.021	1.18	1.02–1.36
	Partial resection	Reference	–	–	–
	Segmentectomy	0.06	0.579	1.06	0.87–1.29
	Left lateral sectionectomy	−0.10	0.445	0.90	0.70–1.17
	Right posterior sectionectomy	−0.02	0.898	0.99	0.78–1.24
	Right anterior sectionectomy	0.52	<0.001	1.68	1.34–2.10
	Left medial sectionectomy	0.15	0.343	1.16	0.85–1.57

	Right hepatectomy	0.56	<0.001	1.75	1.50–2.03
	Left hepatectomy	0.44	<0.001	1.55	1.32–1.82
	Central bisectionectomy	0.67	<0.001	1.95	1.43–2.65
	Right trisectionectomy	0.82	<0.001	2.27	1.50–3.43
	Left trisectionectomy	1.46	<0.001	4.32	2.97–6.27
	Hepatectomy for gallbladder cancer	1.10	<0.001	3.00	2.33–3.86
	Hepatectomy for perihilar cholangiocarcinoma	1.43	<0.001	4.17	3.60–4.83
	No respiratory distress	Reference	–	–	–
	Preoperative respiratory distress	0.37	0.032	1.45	1.03–2.04
	ASA classification 1 and 2	Reference	–	–	–
ASA classification ≥3	0.17	0.008	1.18	1.04–1.34
Surgical site infection (organ space)	Intercept	−4.45	<0.001	0.01	0.01–0.01
	Age	0.05	0.003	1.05	1.02–1.09
	Gender (female)	Reference	–	–	–
	Gender (male)	0.08	0.061	1.08	1.00–1.18
	No preoperative chemotherapy within 90 days before surgery	Reference	–	–	–
	Preoperative chemotherapy within 90 days before surgery	0.23	<0.001	1.26	1.14–1.41
	No smoking within 1 year before surgery	Reference	–	–	–
	Current smoker (within 1 year)	0.09	0.039	1.10	1.00–1.20
	Non drinker	Reference	–	–	–
	Occasional drinker	0.17	<0.001	1.19	1.08–1.30
	Habitual drinker	0.05	0.212	1.06	0.97–1.15
	ADL Independence	Reference	–	–	–
	ADL partial support just before surgery	−0.04	0.717	0.96	0.77–1.20
	ADL full support just before surgery	0.57	0.013	1.78	1.13–2.80
	No COPD	Reference	–	–	–
	COPD	0.14	0.096	1.15	0.98–1.35
	No history of cardiac surgery	Reference	–	–	–
	Previous cardiac surgery	0.23	0.123	1.25	0.94–1.67
	No disseminated cancer	Reference	–	–	–
	Disseminated cancer	0.48	<0.001	1.61	1.37–1.90
	No weight loss	Reference	–	–	–
	Weight loss >10%	0.40	<0.001	1.50	1.24–1.81
	No anticoagulant therapy	Reference	–	–	–
	Anticoagulant therapy	0.15	0.037	1.16	1.01–1.33
	No sepsis	Reference	–	–	–
	Sepsis	0.64	0.004	1.90	1.23–2.95
	Hemoglobin male ≥13.5 g/dL; female ≥11.5 g/dL	Reference	–	–	–
	Hemoglobin male <13.5 g/dL; female <11.5 g/dL	0.13	0.006	1.14	1.04–1.25
	HCT ≥ 37(male) or 32(female)	Reference	–	–	–
	HCT male <37; female <32	0.13	0.012	1.13	1.03–1.25
	PLT ≥150 000/μL	Reference	–	–	–
	PLT <150 000/μL	−0.12	0.011	0.88	0.81–0.97
	ALB ≥4.0 g/dL	Reference	–	–	–
	ALB <4.0 g/dL	0.22	<0.001	1.25	1.15–1.36

	ALP ≤340 U/L	Reference	–	–	–
	ALP >340 U/L	0.27	<0.001	1.31	1.21–1.42
	Serum Na 138–146 mEq/L	Reference	–	–	–
	Serum Na >146 or < 138 mEq/L	0.10	0.079	1.10	0.99–1.22
	CRP ≤0.1 mg/dL	Reference	–	–	–
	CRP >0.1 mg/dL	0.19	<0.001	1.22	1.13–1.31
	PT% ≥70 or PT sec ≤12	Reference	–	–	–
	PT% <70 or PT sec >12	−0.13	0.017	0.87	0.78–0.98
	Partial resection	Reference	–	–	–
	Segmentectomy	0.08	0.325	1.09	0.92–1.28
	Left lateral sectionectomy	−0.33	0.005	0.72	0.57–0.91
	Right posterior sectionectomy	0.32	<0.001	1.38	1.17–1.62
	Right anterior sectionectomy	0.79	<0.001	2.20	1.86–2.60
	Left medial sectionectomy	0.16	0.217	1.17	0.91–1.50
	Right hepatectomy	0.45	<0.001	1.57	1.38–1.78
	Left hepatectomy	0.67	<0.001	1.95	1.72–2.20
	Central bisectionectomy	0.93	<0.001	2.53	2.01–3.19
	Right trisectionectomy	0.80	<0.001	2.22	1.57–3.14
	Left trisectionectomy	1.50	<0.001	4.48	3.29–6.10
	Hepatectomy for gallbladder cancer	1.56	<0.001	4.74	3.97–5.65
	Hepatectomy for perihilar cholangiocarcinoma	1.89	<0.001	6.61	5.91–7.39
	No chronic steroid use	Reference	–	–	–
Chronic steroid use	0.36	0.013	1.43	1.08–1.90
Bile leakage	Intercept	−4.29	<0.001	0.01	0.01–0.02
	Age	0.07	<0.001	1.07	1.04–1.10
	Gender (female)	Reference	–	–	–
	Gender (male)	0.15	<0.001	1.16	1.08–1.25
	Body mass index ≤30 kg/m^2^	Reference	–	–	–
	Body mass index >30 kg/m^2^	−0.13	0.142	0.88	0.74–1.04
	Non drinker	Reference	–	–	–
	Occasional drinker	0.14	<0.001	1.15	1.06–1.24
	Habitual drinker	0.08	0.031	1.08	1.01–1.16
	No angina	Reference	–	–	–
	Angina within 30 d before surgery	−0.33	0.048	0.72	0.52–1.00
	No disseminated cancer	Reference	–	–	–
	Disseminated cancer	0.15	0.069	1.17	0.99–1.38
	No anticoagulant therapy	Reference	–	–	–
	Anticoagulant therapy	0.10	0.095	1.10	0.98–1.24
	No preoperative blood transfusion	Reference	–	–	–
	Preoperative blood transfusion	0.27	0.093	1.31	0.96–1.81
	No tumor	Reference	–	–	–
	Benign tumor	−0.14	0.201	0.87	0.69–1.08
	Malignant tumor	−0.15	0.042	0.86	0.75–0.99
	Hemoglobin male ≥13.5 g/dL; female ≥11.5 g/dL	Reference	–	–	–
	Hemoglobin male <13.5 g/dL; female <11.5 g/dL	0.10	0.004	1.10	1.03–1.17
	PLT ≥150 000/μL	Reference	–	–	–
	PLT <150 000/μL	−0.07	0.06	0.93	0.86–1.00

	ALB ≥4.0 g/dL	Reference	–	–	–
	ALB <4.0 g/dL	0.13	<0.001	1.14	1.07–1.22
	ALP ≤340 U/L	Reference	–	–	–
	ALP >340 U/L	0.31	<0.001	1.37	1.28–1.46
	Creatinine male:0.61–1.04 mg/dL, female:0.47–0.79 mg/dL	Reference	–	–	–
	Creatinine male >1.04 mg/dL or <0.61 mg/dL; female >0.79 mg/dL or <0.47 mg/dL	−0.06	0.11	0.95	0.88–1.01
	CRP ≤0.1 mg/dL	Reference	–	–	–
	CRP >0.1 mg/dL	0.14	<0.001	1.16	1.08–1.23
	Partial resection	Reference	–	–	–
	Segmentectomy	0.80	<0.001	2.22	1.95–2.52
	Left lateral sectionectomy	−0.16	0.163	0.86	0.69–1.07
	Right posterior sectionectomy	0.72	<0.001	2.06	1.78–2.38
	Right anterior sectionectomy	1.72	<0.001	5.60	4.93–6.37
	Left medial sectionectomy	0.91	<0.001	2.47	2.05–2.98
	Right hepatectomy	0.99	<0.001	2.69	2.40–3.01
	Left hepatectomy	1.29	<0.001	3.62	3.25–4.03
	Central bisectionectomy	2.04	<0.001	7.71	6.55–9.07
	Right trisectionectomy	1.55	<0.001	4.69	3.56–6.17
	Left trisectionectomy	2.35	<0.001	10.45	8.18–13.35
	Hepatectomy for gallbladder cancer	2.06	<0.001	7.81	6.70–9.11
	Hepatectomy for perihilar cholangiocarcinoma	2.22	<0.001	9.17	8.26–10.19
	No chronic steroid use	Reference	–	–	–
	Chronic steroid use	0.33	0.009	1.39	1.08–1.78
	Pneumonia Intercept	−8.67	<0.001	0	0.00–0.00
	Age	0.42	<0.001	1.52	1.42–1.64
	Gender (female)	Reference	–	–	–
	Gender (male)	0.66	<0.001	1.94	1.65–2.27
	No smoking within 1 year before surgery	Reference	–	–	–
	Current smoker (within 1 y)	0.22	0.003	1.24	1.08–1.43
	ADL Independence	Reference	–	–	–
	ADL partial support just before surgery	0.66	<0.001	1.94	1.52–2.47
	ADL full support just before surgery	1.15	<0.001	3.16	1.95–5.12
	No COPD	Reference	–	–	–
	COPD	0.52	<0.001	1.68	1.36–2.07
	No pneumonia	Reference	–	–	–
	Pneumonia	2.03	<0.001	7.59	4.57–12.61
	No weight loss	Reference	–	–	–
	Weight loss >10%	0.47	0.001	1.60	1.20–2.13
	No anticoagulant therapy	Reference	–	–	–
	Anticoagulant therapy	0.18	0.073	1.20	0.98–1.45
	No sepsis	Reference	–	–	–
	Sepsis	1.05	<0.001	2.85	1.70–4.77
	WBC 3500–9000/μL	Reference	–	–	–
	WBC <3500/μL	−0.20	0.117	0.81	0.63–1.05
WBC >9000/μL	0.28	0.009	1.33	1.07–1.64

	HCT ≥ 37(male) or 32(female)	Reference	–	–	–
	HCT male <37; female <32	0.18	0.007	1.20	1.05–1.37
	PLT ≥150 000/μL	Reference	–	–	–
	PLT <150 000/μL	0.14	0.051	1.15	1.00–1.33
	ALB ≥4.0 g/dL	Reference	–	–	–
	ALB <4.0 g/dL	0.36	<0.001	1.43	1.25–1.64
	AST ≤35 U/L	Reference	–	–	–
	AST >35 U/L	0.10	0.118	1.11	0.97–1.26
	ALP ≤340 U/L	Reference	–	–	–
	ALP >340 U/L	0.19	0.006	1.21	1.06–1.38
	BUN ≤20 mg/dL	Reference	–	–	–
	BUN >20 mg/dL	−0.15	0.078	0.86	0.72–1.02
	Creatinine male:0.61–1.04 mg/dL, female:0.47–0.79 mg/dL	Reference	–	–	–
	Creatinine male >1.04 mg/dL or <0.61 mg/dL; female >0.79 mg/dL or <0.47 mg/dL	0.17	0.015	1.18	1.03–1.35
	Serum Na 138–146 mEq/L	Reference	–	–	–
	Serum Na >146 or < 138 mEq/L	0.31	<0.001	1.36	1.17–1.59
	CRP ≤0.1 mg/dL	Reference	–	–	–
	CRP >0.1 mg/dL	0.20	0.003	1.22	1.07–1.39
	APTT ≤40 sec	Reference	–	–	–
	APTT >40 sec	0.30	0.007	1.35	1.09–1.68
	No respiratory distress	Reference	–	–	–
	Preoperative respiratory distress	0.64	<0.001	1.89	1.37–2.60
	No history of cerebrovascular disease	Reference	–	–	–
	Previous cerebrovascular disease	0.30	0.01	1.36	1.07–1.71
	ASA classification 1 and 2	Reference	–	–	–
	ASA classification ≥3	0.29	<0.001	1.34	1.16–1.54
	Partial resection	Reference	–	–	–
	Segmentectomy	0.14	0.254	1.15	0.91–1.46
	Left lateral sectionectomy	−0.13	0.437	0.88	0.63–1.22
	Right posterior sectionectomy	0.36	0.004	1.43	1.12–1.83
	Right anterior sectionectomy	0.85	<0.001	2.33	1.83–2.97
	Left medial sectionectomy	0.04	0.825	1.04	0.71–1.54
	Right hepatectomy	0.51	<0.001	1.66	1.37–2.02
	Left hepatectomy	0.22	0.046	1.24	1.00–1.54
	Central bisectionectomy	0.40	0.051	1.50	1.00–2.24
	Trisectionectomy	0.91	<0.001	2.48	1.67–3.69
	Hepatectomy for gallbladder cancer	0.47	0.016	1.60	1.09–2.36
Hepatectomy for perihilar cholangiocarcinoma	0.84	<0.001	2.31	1.88–2.83
Renal failure	Intercept	−7.47	<0.001	0	0.00–0.00
	Age	0.14	<0.001	1.15	1.08–1.23
	Gender (female)	Reference	–	–	–
	Gender (male)	0.53	<0.001	1.69	1.44–1.99
	No preoperative chemotherapy within 90 days before surgery	Reference	–	–	–
	Preoperative chemotherapy within 90 days before surgery	−0.18	0.099	0.83	0.67–1.03
	Body mass index ≤30 kg/m^2^	Reference	–	–	–

	Body mass index >30 kg/m^2^	0.41	0.002	1.51	1.16–1.97
	Non drinker	Reference	–	–	–
	Occasional drinker	0.18	0.02	1.20	1.03–1.40
	Habitual drinker	0.03	0.642	1.03	0.90–1.19
	ADL Independence	Reference	–	–	–
	ADL partial support just before surgery	0.22	0.125	1.24	0.94–1.64
	ADL full support just before surgery	0.59	0.044	1.80	1.02–3.20
	No hypertension	Reference	–	–	–
	Hypertension (untreated)	0.19	0.323	1.21	0.83–1.76
	Hypertension (internal medicine therapy)	0.30	<0.001	1.36	1.20–1.54
	No dialysis	Reference	–	–	–
	Dialysis	0.85	<0.001	2.34	1.77–3.09
	No disseminated cancer	Reference	–	–	–
	Disseminated cancer	0.23	0.139	1.26	0.93–1.71
	No weight loss	Reference	–	–	–
	Weight loss >10%	0.56	<0.001	1.75	1.32–2.31
	No preoperative blood transfusion	Reference	–	–	–
	Preoperative blood transfusion	0.61	0.002	1.84	1.25–2.69
	No sepsis	Reference	–	–	–
	Sepsis	1.18	<0.001	3.24	1.96–5.34
	Hemoglobin male ≥13.5 g/dL; female ≥11.5 g/dL	Reference	–	–	–
	Hemoglobin male <13.5 g/dL; female <11.5 g/dL	0.13	0.12	1.14	0.97–1.35
	HCT ≥ 37(male) or 32(female)	Reference	–	–	–
	HCT male <37; female <32	0.29	<0.001	1.33	1.13–1.56
	PLT ≥150 000/μL	Reference	–	–	–
	PLT <150 000/μL	0.33	<0.001	1.39	1.22–1.59
	ALB ≥4.0 g/dL	Reference	–	–	–
	ALB <4.0 g/dL	0.29	<0.001	1.34	1.16–1.53
	AST ≤35 U/L	Reference	–	–	–
	AST >35 U/L	0.20	0.002	1.22	1.08–1.39
	ALP ≤340 U/L	Reference	–	–	–
	ALP >340 U/L	0.29	<0.001	1.33	1.17–1.52
	BUN ≤20 mg/dL	Reference	–	–	–
	BUN >20 mg/dL	0.54	<0.001	1.72	1.49–1.98
	Creatinine male:0.61–1.04 mg/dL, female:0.47–0.79 mg/dL	Reference	–	–	–
	Creatinine male >1.04 mg/dL or <0.61 mg/dL; female >0.79 mg/dL or <0.47 mg/dL	0.79	<0.001	2.20	1.93–2.50
	PT INR ≤1.1	Reference	–	–	–
	PT INR >1.1	0.15	0.088	1.17	0.98–1.39
	PT% ≥70 or PT sec ≤12	Reference	–	–	–
	PT% <70 or PT sec >12	0.15	0.093	1.16	0.98–1.39
	Partial resection	Reference	–	–	–
	Segmentectomy	0.08	0.575	1.08	0.83–1.41
	Left lateral sectionectomy	−0.12	0.516	0.89	0.61–1.28
	Right posterior sectionectomy	0.63	<0.001	1.87	1.46–2.40
Right anterior sectionectomy	1.15	<0.001	3.15	2.48–4.01

	Left medial sectionectomy	0.16	0.422	1.18	0.79–1.75
	Right hepatectomy	1.06	<0.001	2.90	2.40–3.50
	Left hepatectomy	0.40	<0.001	1.50	1.19–1.89
	Central bisectionectomy	0.94	<0.001	2.57	1.77–3.73
	Right trisectionectomy	1.29	<0.001	3.64	2.24–5.93
	Left trisectionectomy	1.82	<0.001	6.15	3.83–9.88
	Hepatectomy for gallbladder cancer	0.90	<0.001	2.45	1.63–3.70
	Hepatectomy for perihilar cholangiocarcinoma	1.46	<0.001	4.30	3.50–5.28
	No respiratory distress	Reference	–	–	–
	Preoperative respiratory distress	0.57	0.002	1.77	1.24–2.53
	ASA classification 1 and 2	Reference	–	–	–
	ASA classification ≥3	0.28	<0.001	1.32	1.14–1.52
	No diabetes	Reference	–	–	–
	Diabetes (no treatment and dietary treatment only)	0.21	0.114	1.24	0.95–1.61
	Diabetes (internal medicine therapy)	0.18	0.018	1.19	1.03–1.38
Diabetes (insulin therapy)	0.47	<0.001	1.60	1.33–1.94
Postoperative blood transfusion	Intercept	−5.13	<0.001	0.01	0.00–0.01
	Age	0.09	<0.001	1.10	1.06–1.14
	Gender (female)	Reference	–	–	–
	Gender (male)	0.07	0.092	1.08	0.99–1.17
	No preoperative chemotherapy within 90 days before surgery	Reference	–	–	–
	Preoperative chemotherapy within 90 days before surgery	−0.10	0.152	0.90	0.78–1.04
	Body mass index ≤30 kg/m^2^	Reference	–	–	–
	Body mass index >30 kg/m^2^	0.24	0.012	1.27	1.05–1.53
	ADL Independence	Reference	–	–	–
	ADL partial support just before surgery	0.45	<0.001	1.57	1.31–1.88
	ADL full support just before surgery	0.24	0.291	1.27	0.82–1.98
	No history of cardiac surgery	Reference	–	–	–
	Previous cardiac surgery	0.25	0.061	1.29	0.99–1.68
	No dialysis	Reference	–	–	–
	Dialysis	0.24	0.095	1.27	0.96–1.69
	No disseminated cancer	Reference	–	–	–
	Disseminated cancer	0.41	<0.001	1.51	1.27–1.81
	No weight loss	Reference	–	–	–
	Weight loss >10%	0.39	<0.001	1.48	1.22–1.79
	No anticoagulant therapy	Reference	–	–	–
	Anticoagulant therapy	0.11	0.125	1.11	0.97–1.28
	No preoperative blood transfusion	Reference	–	–	–
	Preoperative blood transfusion	0.92	<0.001	2.52	1.96–3.24
	No preoperative chemotherapy within 30 days before surgery	Reference	–	–	–
	Preoperative chemotherapy within 30 days before surgery	0.17	0.135	1.18	0.95–1.48
	No sepsis	Reference	–	–	–
	Sepsis	0.46	0.033	1.59	1.04–2.43
	No tumor	Reference	–	–	–
	Benign tumor	−0.38	0.02	0.69	0.50–0.94
	Malignant tumor	−0.15	0.111	0.86	0.71–1.04

	Hemoglobin male ≥13.5 g/dL; female ≥11.5 g/dL	Reference	–	–	–
	Hemoglobin male <13.5 g/dL; female <11.5 g/dL	0.23	<0.001	1.26	1.14–1.40
	HCT ≥ 37(male) or 32(female)	Reference	–	–	–
	HCT male <37; female <32	0.31	<0.001	1.37	1.23–1.51
	PLT ≥150 000/μL	Reference	–	–	–
	PLT <150 000/μL	0.25	<0.001	1.29	1.18–1.41
	ALB ≥4.0 g/dL	Reference	–	–	–
	ALB <4.0 g/dL	0.18	<0.001	1.19	1.09–1.30
	Total bilirubin ≤1.2 mg/dL	Reference	–	–	–
	Total bilirubin >1.2 mg/dL	0.09	0.155	1.09	0.97–1.23
	AST ≤35 U/L	Reference	–	–	–
	AST >35 U/L	0.19	<0.001	1.21	1.12–1.32
	ALP ≤340 U/L	Reference	–	–	–
	ALP >340 U/L	0.22	<0.001	1.25	1.15–1.36
	Creatinine male:0.61–1.04 mg/dL, female:0.47–0.79 mg/dL	Reference	–	–	–
	Creatinine male >1.04 mg/dL or <0.61 mg/dL; female >0.79 mg/dL or <0.47 mg/dL	0.18	<0.001	1.19	1.10–1.30
	Serum Na 138–146 mEq/L	Reference	–	–	–
	Serum Na >146 or < 138 mEq/L	0.21	<0.001	1.24	1.11–1.37
	CRP ≤0.1 mg/dL	Reference	–	–	–
	CRP >0.1 mg/dL	0.17	<0.001	1.19	1.09–1.29
	PT INR ≤1.1	Reference	–	–	–
	PT INR >1.1	0.16	0.005	1.18	1.05–1.32
	PT% ≥70 or PT sec ≤12	Reference	–	–	–
	PT% <70 or PT sec >12	0.13	0.03	1.13	1.01–1.27
	ASA classification 1 and 2	Reference	–	–	–
	ASA classification ≥3	0.30	<0.001	1.35	1.22–1.48
	No respiratory distress	Reference	–	–	–
	Preoperative respiratory distress	0.24	0.092	1.27	0.96–1.68
	Partial resection	Reference	–	–	–
	Segmentectomy	0.33	<0.001	1.40	1.18–1.65
	Left lateral sectionectomy	0.21	0.051	1.23	1.00–1.52
	Right posterior sectionectomy	0.78	<0.001	2.17	1.85–2.55
	Right anterior sectionectomy	1.24	<0.001	3.44	2.93–4.04
	Left medial sectionectomy	0.30	0.023	1.35	1.04–1.75
	Right hepatectomy	1.10	<0.001	3.00	2.65–3.39
	Left hepatectomy	0.66	<0.001	1.93	1.67–2.22
	Central bisectionectomy	1.21	<0.001	3.34	2.65–4.21
	Right trisectionectomy	1.22	<0.001	3.38	2.44–4.68
	Left trisectionectomy	1.94	<0.001	6.93	5.12–9.37
	Hepatectomy for gallbladder cancer	0.91	<0.001	2.50	1.94–3.20
Hepatectomy for perihilar cholangiocarcinoma	1.49	<0.001	4.43	3.88–5.06
Sepsis	Intercept	−6.7	<0.001	0	0.00–0.00
	Age	0.14	<0.001	1.15	1.09–1.21
	Gender (female)	Reference	–	–	–
	Gender (male)	0.36	<0.001	1.44	1.27–1.63

	No emergency operation	Reference	–	–	–
	Emergency operation	0.44	0.075	1.56	0.96–2.53
	Body mass index ≤30 kg/m^2^	Reference	–	–	–
	Body mass index >30 kg/m^2^	0.42	0.002	1.52	1.17–1.98
	ADL Independence	Reference	–	–	–
	ADL partial support just before surgery	0.30	0.028	1.35	1.03–1.76
	ADL full support just before surgery	0.43	0.163	1.53	0.84–2.78
	No dialysis	Reference	–	–	–
	Dialysis	0.39	0.076	1.48	0.96–2.27
	No disseminated cancer	Reference	–	–	–
	Disseminated cancer	0.57	<0.001	1.76	1.37–2.26
	No weight loss	Reference	–	–	–
	Weight loss >10%	0.65	<0.001	1.92	1.51–2.44
	No preoperative chemotherapy within 30 days before surgery	Reference	–	–	–
	Preoperative chemotherapy within 30 days before surgery	0.25	0.068	1.29	0.98–1.69
	No sepsis	Reference	–	–	–
	Sepsis	1.80	<0.001	6.05	4.04–9.05
	HCT ≥ 37(male) or 32(female)	Reference	–	–	–
	HCT male <37; female <32	0.24	<0.001	1.28	1.13–1.44
	PLT ≥150 000/μL	Reference	–	–	–
	PLT <150 000/μL	0.24	<0.001	1.28	1.12–1.46
	ALB ≥4.0 g/dL	Reference	–	–	–
	ALB <4.0 g/dL	0.33	<0.001	1.40	1.23–1.58
	AST ≤35 U/L	Reference	–	–	–
	AST >35 U/L	0.14	0.017	1.15	1.02–1.29
	ALP ≤340 U/L	Reference	–	–	–
	ALP >340 U/L	0.35	<0.001	1.42	1.26–1.60
	Creatinine male:0.61–1.04 mg/dL, female:0.47–0.79 mg/dL	Reference	–	–	–
	Creatinine male >1.04 mg/dL or <0.61 mg/dL; female >0.79 mg/dL or <0.47 mg/dL	0.11	0.069	1.12	0.99–1.26
	Serum Na 138–146 mEq/L	Reference	–	–	–
	Serum Na >146 or < 138 mEq/L	0.17	0.025	1.18	1.02–1.37
	CRP ≤0.1 mg/dL	Reference	–	–	–
	CRP >0.1 mg/dL	0.13	0.026	1.14	1.02–1.29
	APTT ≤40 sec	Reference	–	–	–
	APTT >40 sec	0.24	0.028	1.27	1.03–1.58
	PT% ≥70 or PT sec ≤12	Reference	–	–	–
	PT% <70 or PT sec >12	0.11	0.145	1.12	0.96–1.30
	Partial resection	Reference	–	–	–
	Segmentectomy	0.23	0.094	1.26	0.96–1.66
	Left lateral sectionectomy	0.23	0.175	1.26	0.90–1.76
	Right posterior sectionectomy	0.37	0.013	1.44	1.08–1.93
	Right anterior sectionectomy	1.22	<0.001	3.39	2.64–4.36
	Left medial sectionectomy	0.32	0.129	1.37	0.91–2.07
	Right hepatectomy	1.05	<0.001	2.85	2.35–3.46
	Left hepatectomy	0.93	<0.001	2.54	2.07–3.11
	Central bisectionectomy	1.25	<0.001	3.49	2.47–4.93
Right trisectionectomy	1.39	<0.001	4.01	2.55–6.31

	Left trisectionectomy	2.11	<0.001	8.26	5.48–12.47
	Hepatectomy for gallbladder cancer	1.55	<0.001	4.71	3.46–6.42
	Hepatectomy for perihilar cholangiocarcinoma	2.17	<0.001	8.74	7.29–10.47
	No chronic steroid use	Reference	–	–	–
	Chronic steroid use	0.52	0.009	1.69	1.14–2.50
	ASA classification 1 and 2	Reference	–	–	–
	ASA classification ≥3	0.15	0.046	1.16	1.00–1.34
	No respiratory distress	Reference	–	–	–
Preoperative respiratory distress	0.72	<0.001	2.06	1.47–2.88

*Note*: Age is the value divided by 10.

Abbreviations: ADL, Activities of daily living; ASA, American Society of Anesthesiologists; CI, confidence interval; COPD, chronic obstructive pulmonary disease.

### 3.5 Model performance

**FIGURE 2:**
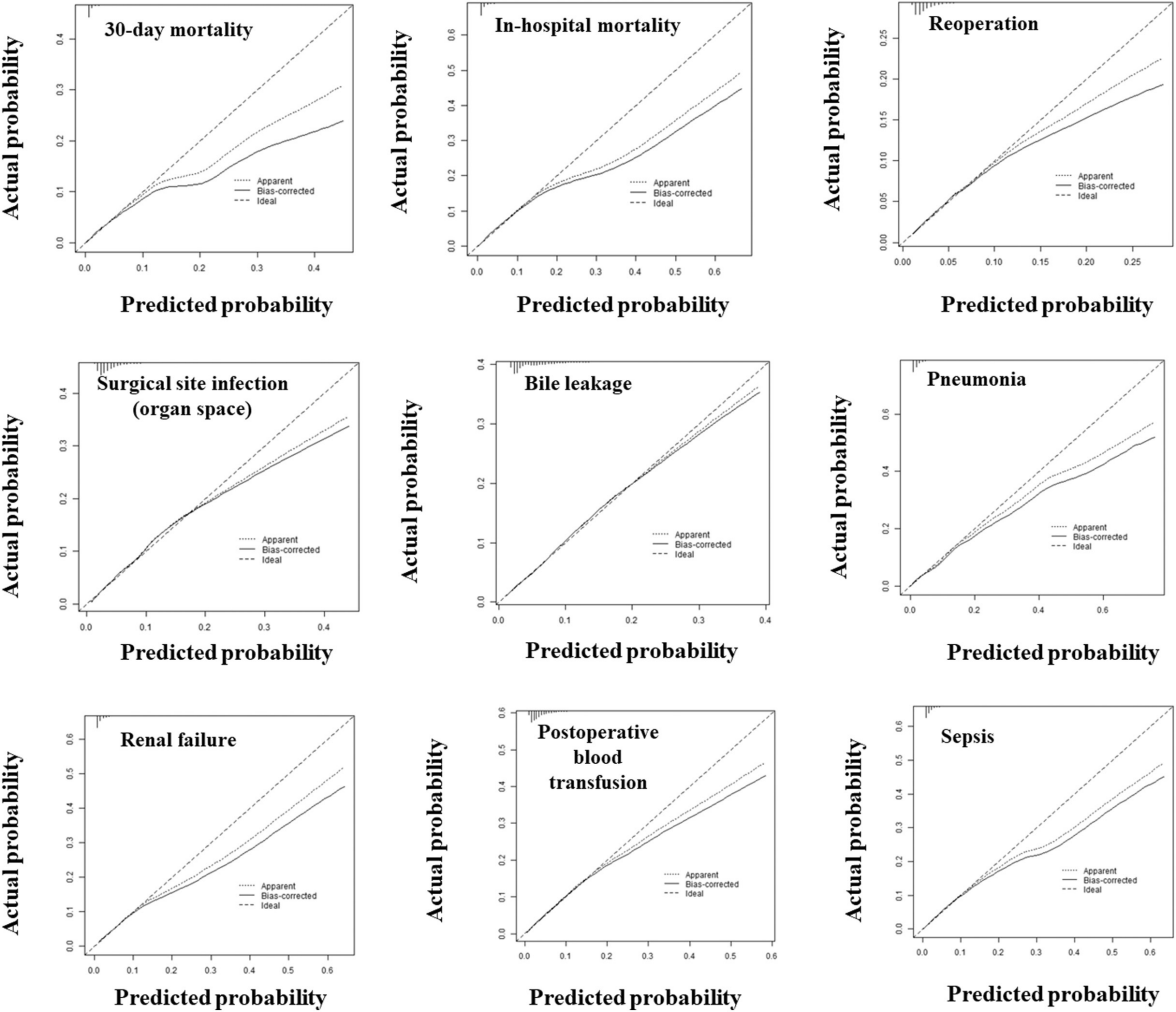
Calibration curves of the risk model showing the predicted vs. actual probability for 30‐day mortality, in‐hospital mortality, reoperation, surgical site infection (organ space), bile leakage, pneumonia, renal failure, postoperative blood transfusion, and sepsis.

The model was validated internally using bootstrapping. The c‐index and 95% CI for each outcome are shown in Table 5. The calibration curves of the models are shown in Figure 2, which also shows the values for 30‐day mortality, in‐hospital mortality, reoperation, surgical site infection (organ space), bile leakage, pneumonia, renal failure, postoperative blood transfusion, and sepsis. These calibration curves indicated how well the predicted event rates match the observed rates among patient risk groups. The calibration plot showed that, for many models, the line deviates from the ideal straight line, except where the prediction probability is low, and overestimates where the prediction probability is high, as indicated in Figure 2. For outcomes with a particularly low number of events, such as 30‐day mortality and in‐hospital mortality, the calibration plot deviates from the ideal straight line, except where the calibration plot has a low predicted probability. However, for outcomes with a relatively high probability of occurrence, such as biliary leakage, the calibration plot was in good agreement with the ideal straight line. In contrast, the c‐indices for 30 days mortality and in‐hospital mortality were 0.824 and 0.839, respectively, indicating good performance with respect to the outcomes with a low number of events.

**TABLE 5:** C‐indexes across the risk model.

Outcome	C‐index	95% CI
30 days mortality	0.824	0.815–0.841
In‐hospital mortality	0.839	0.831–0.850
Reoperation	0.686	0.680–0.702
Surgical site infection (Organ space)	0.724	0.718–0.733
Bile leakage	0.739	0.734–0.747
Pneumonia	0.750	0.743–0.768
Renal failure	0.803	0.796–0.816
Postoperative blood transfusion	0.732	0.727–0.743
Sepsis	0.774	0.767–0.789

Abbreviation: CI, confidence interval.

### 3.6 Thirty‐day mortality and in‐hospital mortality by number of cases per facility

**FIGURE 3:**
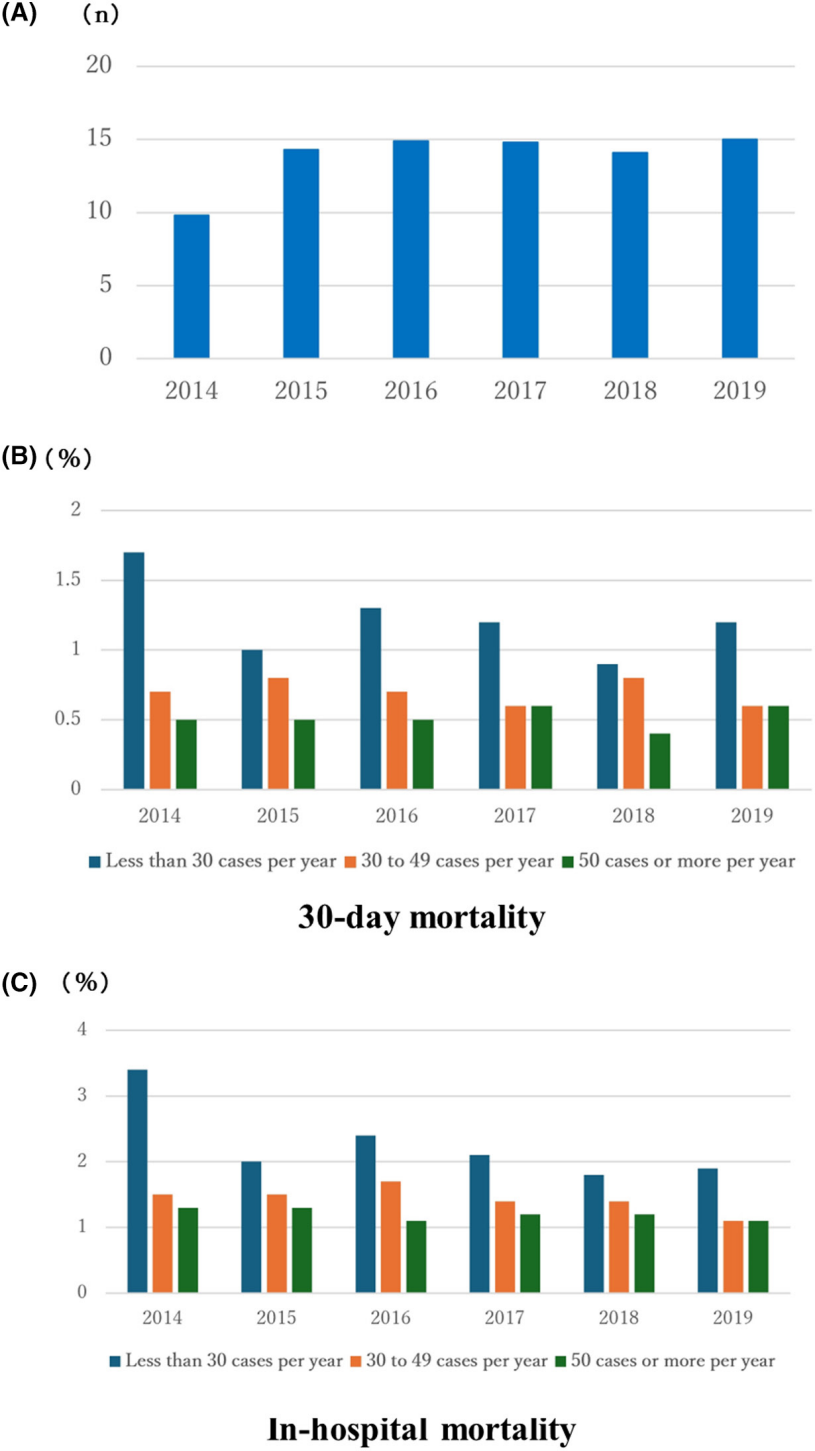
(A) Average number of cases per facility in each year. (B) 30‐day mortality rates by facility size. (C) In‐hospital mortality rates by facility size.

Figure 3a shows the average number of cases per facility in each year. Figure 3b,c show the 30‐day and in‐hospital mortality rates by facility size, respectively. We examined the 30‐day and in‐hospital mortality rates by institution size (i.e. less than 30 cases per year, 30–49 cases per year, or 50 cases or more per year) based on the number of liver resections performed per year (Figure 3b,c). As shown in Figure 3b,c, facilities that conducted 30–49 and 50 or more hepatectomies per year tended to have lower 30‐day and in‐hospital mortality rates than those that had fewer than 30 hepatectomies in any year over the 2014 to 2019 study period. In a comparison of facilities with 30–49 against those with 50 or more liver resections per year, there were years in which the 30‐day and in‐hospital mortality rates were equivalent and years when this rate was lower in facilities that had conducted 50 or more procedures.

## 4 DISCUSSION

Here, we examined more than 70 000 liver resections performed at Japanese centers over a 6‐year period and analyzed the risks associated with a liver resection procedure, based on a national Japanese database. In our analyses, the 30‐day mortality rate was 0.9%, and the in‐hospital mortality rate was 1.7% in the study population. These values were lower than those described in previous NCD reports from Japan.^10^, ^11^ Furthermore, the mortality rates obtained in our current study are lower than those reported by other national databases.^2^, ^3^ This indicated that good surgical standards were maintained in Japan during the study period. We also developed a risk model for liver resection using the Japanese NCD with good‐quality assurance.

We observed that the statistically significant risk factors with an odds ratio of three times or more for 30‐day mortality in our study population were trisectionectomy, hepatectomy for gallbladder cancer, and hepatectomy for perihilar cholangiocarcinoma. Those for in‐hospital mortality were right hepatectomy, right trisectionectomy, left trisectionectomy, hepatectomy for gallbladder cancer, and hepatectomy for perihilar cholangiocarcinoma.

Trisectionectomy and hepatectomy for perihilar cholangiocarcinoma had the highest odds ratios. These variables have been reported as risk factors for liver resection in previous studies. Lang et al. reported an operative mortality rate of 11.9 % associated with left trisectionectomy.^13^ Farid reported a 90‐day mortality rate from left trisectionectomy of 9.7%.^14^ Recently, Kron et al. reported an overall 90‐day mortality rate of 7.6 % from trisectionectomy for hepatopancreatobiliary malignancies.^15^ A high perioperative mortality has been similarly reported following surgery for perihilar cholangiocarcinoma, for which Mueller et al. reported median in‐hospital and 3‐month mortality rates of 4.7% and 7%, respectively.^16^ Recently, the Perihilar Cholangiocarcinoma Collaboration Group reported a 90‐day mortality rate of 13.6% for perihilar cholangiocarcinoma using an international database.^17^ Perioperative mortality rates >10% for perihilar cholangiocarcinoma have been reported elsewhere.^18^ Similarly, 90‐day mortality rates of more than 10% have been reported for gallbladder cancer.^19^

The risk factors for perioperative mortality in liver surgery identified in the present study are consistent with previous reports. In contrast, full ADL support within 30 days prior to surgery was identified as a risk factor for both 30‐day mortality and in‐hospital mortality.

In addition to the liver resection protocol, poor capacity to tolerate surgery can also increase the risk of surgery‐related death. Previous reports indicated a strong correlation between frailty and postoperative mortality.^20^, ^21^, ^22^ Our current results are consistent with those of previous reports on patient‐related risk factors. Collectively, these findings indicate that massive major hepatectomy, liver resection with biliary reconstruction, and assisted ADL should be recognized as potential risk factors for hepatectomy‐related death. The present study was also based on information from a national database and an extremely large sample size, which contributes to the reliability of the findings.

Here, we analyzed the risk factors not only for operation‐related mortality but also for postoperative complications that arise following liver resection. Regarding postoperative adverse events, the type of liver resection was a statistically significant risk factor, with a high odds ratio. Similar to mortality, trisectionectomy, hepatectomy for gallbladder cancer, and hepatectomy for perihilar cholangiocarcinoma were identified as risk factors of various postoperative complications in the present study. Farid reported that postoperative morbidity arising from left trisectionectomy ranges from 45% to 50%.^14^ Kron et al. found that 40.3% of patients who underwent right trisectionectomy had postoperative complications.^15^ Mueller et al. reported an overall postoperative complication rate of 80.5% after surgery for perihilar cholangiocarcinoma, with severe complications (grade ≥IIIa) occurring in 58.1% of these patients.^16^ Other hepatectomy procedures with a large liver dissection area, such as right anterior sectionectomy and central bisectionectomy, were also observed in the present study cohort to be a risk factor for bile leakage and other adverse events. Other prior studies have also demonstrated that right anterior sectionectomy and central bisectionectomy are risk factors for bile leakage.^23^, ^24^ On the other hand, the patient‐side risk factors identified herein for postoperative complications with odds ratios of three or more times were full ADL support within 30 days before surgery, preoperative pneumonia, and preoperative sepsis.

Thus, the need for assisted ADL may be a risk factor for complications after hepatectomy as well as a risk factor for surgery‐related death following this procedure. Our results are thus consistent with the findings of previous reports that have shown that frailty is a strong predictor of adverse outcomes from postoperative complications as well as postoperative mortality in surgical patients.^20^, ^21^, ^22^ In addition, preoperative infections such as pneumonia or sepsis can be risk factors for post‐hepatectomy complications. Therefore, massive major hepatectomy, liver resection with biliary reconstruction, hepatectomy with a large liver dissection area, such as a right anterior sectionectomy or central bisectionectomy, fully assisted ADL, and preoperative infection events should be recognized as potential risk factors for hepatectomy‐related postoperative complications.

The C‐indices of 30‐day mortality and in‐hospital mortality in this present study were 0.824 and 0.839, respectively (Table 5). The C‐indices of bile leakage, pneumonia, renal failure, and sepsis in this study were 0.739, 0.750, 0.803, and 0.774, respectively (Table 5). In a previous analysis of Japanese NCD data, Kenjo et al. reported that the C‐indices of 30‐ and 90‐day in‐hospital mortality were 0.714 and 0.761, respectively.^10^ Similarly, with regard to postoperative complications, Yokoo et al. reported that the C‐indices of bile leakage, pneumonia, renal failure, and sepsis were 0.635, 0.749, 0.756, and 0.784, respectively, in a previous study of Japanese NCD data.^11^ Our current results thus appear to have superior prediction accuracy for both morbidity and mortality except for sepsis. It must be noted of course that the validity of this comparison is impacted by differences in the data analyzed. However, our present results suggest that the risk model we have formulated in this current study for both morbidity and mortality may be reliable in clinical practice.

We found from our current analyses that the hospital volume affected both the 30‐day and in‐hospital mortality. It is known that the degree of correlation between hospital volume and surgical mortality varies significantly among surgical procedures, and the concept of "failure to rescue" has recently gained attention with regard to postoperative mortality.^25^, ^26^ Postoperative morbidity rates are similar between hospitals with low mortality and those with high mortality, but there is a significant difference in the success rate of postoperative management (i.e., "failure to rescue"), which is believed to be related to mortality rates. It should be recognized therefore that in addition to the preoperative factors examined in this study, "failure to rescue" can also influence mortality.

The present study had some notable limitations. First, it was limited to the Japanese population. Validation using databases from other countries is necessary to evaluate the general applicability of the results. Second, although we analyzed the procedures used for liver resection in our large cohort, we did not stratify these cases by open or laparoscopic surgery. To ensure better comparisons between the centers represented by our study population and to try and more fairly reflect the outcomes, our approach was to assess risk in accordance with patient background factors and not to include surgeon‐selectable factors or perioperative events that were not known preoperatively among the covariates. In addition, we did not include institutional factors in the risk model variables. Previous reports have shown that center effects can also influence outcomes in the field of hepatobiliary surgery.^27^, ^28^ We here evaluated the risk of all hepatectomies based on patient background factors alone. The reason for this was that our risk model aimed to compare outcomes at different centers based solely on preoperative patient factors. The inclusion of center effects as a predictor variable seems to be contrary to a fair comparison between facilities. It should be recognized, however, that center effects may influence outcomes. Third, for outcomes with a small number of events, the predicted probability tended to overestimate the actual probabilities. Hence, surgeons should be aware of the possibility of overestimation of outcomes with a low number of events.

In conclusion, we examined more than 70 000 Japanese liver resection cases using a nationwide surgical database and developed a risk model for these operations. This model can predict 30‐day mortality, in‐hospital mortality, and major and life‐threatening postoperative morbidities after liver resection, based on preoperative factors alone. Hence, it can assist surgeons and patients in better understanding the risks of these surgeries and making appropriate preoperative decisions.

## FUNDING INFORMATION

This work was supported by the Japanese Society of Gastroenterological Surgery.

## CONFLICT OF INTEREST STATEMENT

Hiroaki Miyata is affiliated with the Department of Healthcare Quality Assessment at the University of Tokyo. The department is a social collaboration department supported by grants from the National Clinical Database, Johnson & Johnson K.K, Nipro Corporation, and Intuitive Surgical Sàrl. Shinya Hirakawa and Hisateru Tachimori belong to an endowed course of Keio University funded by Takeda Pharmaceutical Company Limited and belong to the Department of Healthcare Quality Assessment at the University of Tokyo that accepts financial support from National Clinical Database, Johnson & Johnson K.K., Nipro Corporation, and Intuitive Surgical Sàrl. But Dr Miyata, Dr Hirakawa, and Dr Tachimori have no conflicts of interest regarding this research. The other authors declare no conflicts of interest regarding this research. Dr. Yoshihiro Kakeji and Dr. Ken Shirabe are editorial members of *Annals of Gastroenterological Surgery*.

## ETHICS STATEMENTS

The Ethics Committee of the NCD approved the retrospective use of data collected by the NCD for observational research.

Informed Consent: N/A

Registry and the Registration No. of the study/Trial: N/A

Animal Studies: N/A

## References

[1] Dimick JB , Wainess RM , Cowan JA , Upchurch GR Jr , Knol JA . Colletti LM.J improving perioperative outcome expands the role of hepatectomy in management of benign and malignant hepatobiliary diseases: analysis of 1222 consecutive patients from a prospective database. J Am Coll Surg. 2004;199:31–38. 15383797 10.1097/01.sla.0000141195.66155.0c PMC1356471

[2] He J , Amini N , Spolverato G , Hirose K , Makary M , Wolfgang CL , et al. National trends with a laparoscopic liver resection: results from a population‐based analysis. HPB (Oxford). 2015;17:919–926. 26234323 10.1111/hpb.12469 PMC4571760

[3] Aloia TA , Fahy BN , Fischer CP , Jones SL , Duchini A , Galati J , et al. Predicting poor outcome following hepatectomy: analysis of 2313 hepatectomies in the NSQIP database. HPB (Oxford). 2009;11(6):510–515. 19816616 10.1111/j.1477-2574.2009.00095.x PMC2756639

[4] Sasaki A , Tachimori H , Akiyama Y , Oshikiri T , Miyata H , Kakeji Y , et al. Risk model for mortality associated with esophagectomy via a thoracic approach based on data from the Japanese National Clinical Database on malignant esophageal tumors. Surg Today. 2023;53(1):73–81. 35882654 10.1007/s00595-022-02548-x

[5] Kikuchi H , Endo H , Yamamoto H , Ozawa S , Miyata H , Kakeji Y , et al. Impact of reconstruction route on postoperative morbidity after esophagectomy: analysis of esophagectomies in the Japanese National Clinical Database. Ann Gastroenterol Surg. 2021;6(1):46–53. 35106414 10.1002/ags3.12501 PMC8786683

[6] Fujiya K , Kumamaru H , Fujiwara Y , Miyata H , Tsuburaya A , Kodera Y , et al. Preoperative risk factors for postoperative intra‐abdominal infectious complication after gastrectomy for gastric cancer using a Japanese web‐based nationwide database. Gastric Cancer. 2021;24(1):205–213. 32440807 10.1007/s10120-020-01083-3

[7] Kimura W , Miyata H , Gotoh M , Hirai I , Kenjo A , Kitagawa Y , et al. A pancreaticoduodenectomy risk model derived from 8575 cases from a national single‐race population (Japanese) using a web‐based data entry system: the 30‐day and in‐hospital mortality rates for pancreaticoduodenectomy. Ann Surg. 2014;259(4):773–780. 24253151 10.1097/SLA.0000000000000263

[8] Uemura S , Endo H , Ichihara N , Miyata H , Maeda H , Hasegawa H , et al. Day of surgery and mortality after pancreatoduodenectomy: a retrospective analysis of 29 270 surgical cases of pancreatic head cancer from Japan. J Hepatobiliary Pancreat Sci. 2022;29(7):778–784. 34496150 10.1002/jhbp.1043

[9] Mizuma M , Yamamoto H , Miyata H , Gotoh M , Unno M , Shimosegawa T , et al. Impact of a board certification system and implementation of clinical practice guidelines for pancreatic cancer on mortality of pancreaticoduodenectomy. Surg Today. 2020;50(10):1297–1307. 32382777 10.1007/s00595-020-02017-3 PMC7501122

[10] Kenjo A , Miyata H , Gotoh M , Kitagawa Y , Shimada M , Baba H , et al. Risk stratification of 7,732 hepatectomy cases in 2011 from the National Clinical Database for Japan. J Am Coll Surg. 2014;218(3):412–422. 24468222 10.1016/j.jamcollsurg.2013.11.007

[11] Yokoo H , Miyata H , Konno H , Taketomi A , Kakisaka T , Hirahara N , et al. Models predicting the risks of six life‐ threatening morbidities and bile leakage in 14,970 hepatectomy patients registered in the National Clinical Database of Japan. Medicine (Baltimore). 2016;95(49):e5466. 27930526 10.1097/MD.0000000000005466 PMC5265998

[12] Noma H , Shinozaki T , Iba K , Teramukai S , Furukawa TA . Confidence intervals of prediction accuracy measures for multivariable prediction models based on the bootstrap‐ based optimism correction methods. Stat Med. 2021;40(26):5691–5701. 34302372 10.1002/sim.9148

[13] Lang H , Sotiropoulos GC , Brokalaki EI , Radtke A , Frilling A , Molmenti EP , et al. Left hepatic trisectionectomy for hepatobiliary malignancies. J Am Coll Surg. 2006;203(3):311–321. 16931303 10.1016/j.jamcollsurg.2006.05.290

[14] Farid SG , White A , Khan N , Toogood GJ , Prasad KR , Lodge JP . Clinical outcomes of left hepatic trisectionectomy for hepatobiliary malignancy. Br J Surg. 2016;103(3):249–256. 26695377 10.1002/bjs.10059

[15] Kron P , Kimura N , Farid S , Lodge JPA . Current role of trisectionectomy for hepatopancreatobiliary malignancies. Ann Gastroenterol Surg. 2019;3(6):606–619. 31788649 10.1002/ags3.12292 PMC6875946

[16] Mueller M , Breuer E , Mizuno T , Bartsch F , Ratti F , Benzing C , et al. Perihilar cholangiocarcinoma ‐ novel benchmark values for surgical and oncological outcomes from 24 expert centers. Ann Surg. 2021;274(5):780–788. 34334638 10.1097/SLA.0000000000005103

[17] van Keulen AM , Buettner S , Erdmann JI , Pratschke J , Ratti F , Jarnagin WR , et al. Multivariable prediction model for both 90‐day mortality and long‐term survival for individual patients with perihilar cholangiocarcinoma: does the predicted survival justify the surgical risk? Br J Surg. 2023;110(5):599–605. 36918735 10.1093/bjs/znad057 PMC10364519

[18] Wiggers JK , Groot Koerkamp B , Cieslak KP , Doussot A , van Klaveren D , Allen PJ , et al. Postoperative mortality after liver resection for perihilar cholangiocarcinoma: development of a risk score and importance of biliary drainage of the future liver remnant. J Am Coll Surg. 2016;223(2):321–331. 27063572 10.1016/j.jamcollsurg.2016.03.035 PMC4961586

[19] Goussous N , Hosseini M , Sill AM , Cunningham SC . Minimally invasive and open gallbladder cancer resections: 30‐ vs 90‐day mortality. Hepatobiliary Pancreat Dis Int. 2017;16(4):405–411. 28823371 10.1016/S1499-3872(17)60025-0

[20] Lin HS , Watts JN , Peel NM , Hubbard RE . Frailty and post‐operative outcomes in older surgical patients: a systematic review. BMC Geriatr. 2016;16(1):157. 27580947 10.1186/s12877-016-0329-8 PMC5007853

[21] McIsaac DI , Jen T , Mookerji N , Patel A , Lalu MM . Interventions to improve the outcomes of frail people having surgery: a systematic review. PloS One. 2017;12(12):e0190071. 29287123 10.1371/journal.pone.0190071 PMC5747432

[22] Shaw JF , Budiansky D , Sharif F , McIsaac DI . The Association of Frailty with outcomes after cancer surgery: a systematic review and Metaanalysis. Ann Surg Oncol. 2022;29(8):4690–4704. 35072860 10.1245/s10434-021-11321-2

[23] Yamashita Y , Hamatsu T , Rikimaru T , Tanaka S , Shirabe K , Shimada M , et al. Bile leakage after hepatic resection. Ann Surg. 2001;233(1):45–50. 11141224 10.1097/00000658-200101000-00008 PMC1421165

[24] Yamashita YI , Yamamoto H , Miyata H , Kakeji Y , Kitagawa Y , Yamaue H , et al. Risk factors for bile leakage: latest analysis of 10 102 hepatectomies for hepatocellular carcinoma from the Japanese national clinical database. J Hepatobiliary Pancreat Sci. 2021;28(7):556–562. 32897639 10.1002/jhbp.827

[25] Ghaferi AA , Birkmeyer JD , Dimick JB . Complications, failure to rescue, and mortality with major inpatient surgery in medicare patients. Ann Surg. 2009;250(6):1029–1034. 19953723 10.1097/sla.0b013e3181bef697

[26] Endo I , Hirahara N , Miyata H , Yamamoto H , Matsuyama R , Kumamoto T , et al. Mortality, morbidity, and failure to rescue in hepatopancreatoduodenectomy: an analysis of patients registered in the National Clinical Database in Japan. J Hepatobiliary Pancreat Sci. 2021;28(4):305–316. 33609319 10.1002/jhbp.918

[27] Mise Y , Hirakawa S , Tachimori H , Kakeji Y , Kitagawa Y , Komatsu S , et al. Volume‐ and quality‐controlled certification system promotes centralization of complex hepato‐pancreatic‐biliary surgery. J Hepatobiliary Pancreat Sci. 2023;30(7):851–862. 36706938 10.1002/jhbp.1307

[28] Otsubo T , Kobayashi S , Sano K , Misawa T , Katagiri S , Nakayama H , et al. A nationwide certification system to increase the safety of highly advanced hepatobiliary‐pancreatic surgery. J Hepatobiliary Pancreat Sci. 2023;30(1):60–71. 35611453 10.1002/jhbp.1186

